# Single-Cell Multi-Tissue T Cell Clonal Dynamics Reveal Distinct Immune Coercion Landscapes in MSI and MSS Colorectal Cancer

**DOI:** 10.3390/ijms27062689

**Published:** 2026-03-16

**Authors:** Qianhe Zhan, Siwen Zhang, Bofu Cao, Lanming Chen, Lu Xie

**Affiliations:** 1 College of Food Science and Technology, Shanghai Ocean University, Shanghai 201306, China; 2 Shanghai-MOST Key Laboratory of Health and Disease Genomics, Shanghai Institute for Biomedical and Pharmaceutical Technologies, Shanghai 200237, China; 3 School of Public Health, Fudan University, Shanghai 200237, China; 4 School of Health Science and Engineering, University of Shanghai for Science and Technology, Shanghai 200093, China

**Keywords:** colorectal cancer, single-cell sequencing, TCR reconstruction, immune coercion, therapy response

## Abstract

The efficacy of immunotherapy in colorectal cancer (CRC) has long been considered to be closely associated with microsatellite instability (MSI) status. Patients with microsatellite stable (MSS) tumors typically exhibit poor responses to PD-1/PD-L1 inhibitors and a poor prognosis, often being categorized as immunologically ‘cold’ tumors. However, some MSS patients can still achieve favorable therapeutic responses, sometimes even surpassing those of certain MSI patients. Immune-cold and immune-hot tumor phenotypes are largely determined by the abundance, clonal expansion, and functional states of tumor-infiltrating T cells. This suggests that immunotherapy responses are driven by dynamic remodeling of T-cell clonality rather than by MSI status alone. To elucidate the underlying T cell clonal dynamics, integrated single-cell transcriptome (scRNA-seq) and T cell receptor sequencing (scTCR-seq) data analyses from 43 blood and tissue samples of MSI and MSS colorectal cancer patients before and after anti-PD-1 therapy were performed. Using our developed TCR reconstruction pipeline (TORBiT), we systematically analyzed the clonal architecture of the TCR repertoire, inter-tissue migration, and its association with T-cell functional state transitions. From a TCR clonal kinetic perspective, we revealed two distinct modes of immune Coercion that may further affect the immune response: a “high-fluctuation, deep-exhaustion” pattern in MSI tumors and a “high-baseline, strong-suppression” pattern in MSS tumors. These findings provide a novel theoretical foundation and research perspective for understanding the responsiveness and resistance mechanisms to immune checkpoint inhibitors.

## 1. Introduction

Colorectal cancer (CRC) ranks among the leading malignancies worldwide in terms of both incidence and mortality, with over 1.9 million new cases and approximately 900,000 deaths annually [1]. Despite advances in screening and targeted therapies, the 5-year survival rate for metastatic colorectal cancer remains below 15% [2], underscoring the persistent need for optimized treatment strategies [3,4]. CRC exhibits pronounced molecular and biological heterogeneity, among which microsatellite instability (MSI) represents one of the most clinically significant molecular subtypes, exerting a profound impact on tumor immune characteristics and therapeutic responses [4,5]. MSI primarily arises from defects in the DNA mismatch repair (MMR) system, leading to compromised genomic stability. Accordingly, tumors with deficient mismatch repair are classified as dMMR, whereas those with intact MMR function and stable microsatellites are defined as proficient MMR (pMMR) or microsatellite stable (MSS) tumors [5,6,7,8]. Clinically, approximately 15% of sporadic colorectal cancers and the majority of Lynch syndrome-associated tumors display MSI features, while the remaining ~85% of cases are classified as MSS [5,9,10]. Accumulating evidence indicates that MSI colorectal cancers are typically associated with a more immunologically active tumor microenvironment, characterized by increased T-cell infiltration, elevated expression of immune-related genes, and enhanced immune activation signaling. These features have rendered MSI CRC one of the earliest colorectal cancer subtypes shown to exhibit a pronounced clinical response to immune checkpoint inhibitors, thereby driving the exploration and application of immunotherapy in this disease setting [11].

Therapies targeting the PD-1/PD-L1 pathway have achieved remarkable success in colorectal cancer with dMMR/MSI-H features, as well as in other solid tumors [12]. This therapeutic efficacy is commonly attributed to the high mutational burden of MSI-H tumors, which generates abundant neoantigens [5], thereby eliciting pre-existing effector T cell-mediated anti-tumor immune responses [13]. However, with deeper clinical experience, the simplistic paradigm that “MSI-H equals immunotherapy sensitivity” is increasingly challenged [12,14,15,16]. Studies have shown that not all MSI-H patients derive durable benefit from treatment, revealing significant response heterogeneity within this subgroup. Concurrently, encouraging treatment responses have been observed in a subset of MSS patients [17,18,19], with some cases even demonstrating superior efficacy compared to MSI-H patients [15]. As the core effector cells of immune checkpoint blockade (ICB), the functional state of T cells directly dictates the success of anti-tumor responses. The incomplete concordance between MSI status and clinical benefit indicates that MSI merely provides a potential for immune activation, whereas the true therapeutic bottleneck lies in whether T cells can effectively infiltrate tumors, undergo clonal expansion, and sustain their functional capacity within the tumor microenvironment [20]. These observations suggest that the key determinants of immunotherapy response may extend beyond MSI status itself and are more deeply rooted in the precise shaping and fate regulation of T cell clones by the tumor immune microenvironment. Notably, T cell-mediated antitumor immunity is not confined to the tumor site but instead operates within a dynamic, cross-tissue immune network. Previous studies have demonstrated that during immunotherapy for colorectal cancer, the expansion of intratumoral T-cell clones is often closely accompanied by synchronous changes in related clones in the peripheral blood, highlighting a critical role for cross-tissue clonal trafficking and selection in shaping therapeutic responses [21]. However, most existing studies focus on single tissue compartments, making it difficult to distinguish, at the clonal level, between local expansion, peripheral replenishment, and functional state transitions. This limitation hampers a systematic understanding of the dynamic mechanisms underlying responses to immunotherapy [22].

The T-cell receptor (TCR) serves as the key molecule enabling T cells to specifically recognize antigens and initiate adaptive immune responses. The diversity in its structure and function forms the core foundation of anti-tumor immunity. Composed of α and β chains (or γδ chains), TCRs generate enormous diversity through V(D)J recombination, thereby conferring upon T cells the ability to recognize a nearly unlimited array of antigens [23]. Within the tumor microenvironment (TME), TCRs recognize tumor antigen peptides presented by major histocompatibility complex (MHC) molecules, activating T cells and directing their cytotoxic functions [24]. Consequently, the TCR acts not only as the initiating hub of the immune response but also as a crucial bridge linking tumor antigens to T-cell effector functions. In recent years, advances in high-throughput sequencing technologies have established scTCR sequencing (scTCR-Seq) as a powerful tool for dissecting T-cell clonal composition, tracking the dynamics of specific clones, and assessing the quality of immune responses [23,24]. However, reliance on TCR sequence information alone remains insufficient to comprehensively characterize the functional states of T cells within the tumor microenvironment. An increasing body of evidence indicates that integrating TCR clonal information with single-cell transcriptomic data offers indispensable value for resolving the transcriptional features of the same clone across distinct differentiation stages, functional states, or immune stress conditions [25]. Nevertheless, in multi-tissue and longitudinal single-cell study settings, the stable and accurate reconstruction of full-length TCR sequences from transcriptomic data, together with their reliable pairing to cellular functional states, remains a major technical bottleneck limiting the widespread application of this strategy [26]. In this context, methods capable of directly reconstructing complete TCR sequences from single-cell RNA-sequencing data have emerged as one of the key avenues for enabling integrated analyses of TCR clonality and transcriptional states.

At present, multiple computational tools for TCR reconstruction from transcriptomic data have been widely applied, including DERR [27], Cell Ranger VDJ, MiXCR [28], and TRUST4 [29]. These methods have achieved substantial improvements in sensitivity and accuracy, enabling systematic analyses of T-cell clonality without the need for additional dedicated TCR sequencing. However, a considerable proportion of the TCR sequences assembled by current approaches are still classified as non-full-length chains, which imposes clear limitations in research contexts requiring precise α/β chain pairing, TCR structural modeling, or functional validation. For studies centered on integrated TCR-transcriptome analyses, this limitation to some extent constrains the depth of fine-grained clonal resolution and functional association in downstream analyses.

To address these research gaps, we integrated multi-tissue, longitudinal, and paired single-cell data from patients with MSI and MSS colorectal cancer before and after anti-PD-1 therapy, and established a systematic analytical framework. First, by optimizing the TCR reconstruction pipeline, we precisely characterized baseline TCR repertoire features to delineate fundamental differences between MSI and MSS tumors. Second, by analyzing patterns of clonotype sharing, we investigated treatment-driven T-cell selection dynamics. We then mapped clonal dynamics onto specific T-cell functional subsets to elucidate functional state transitions accompanying clonal expansion or contraction. Finally, we evaluated the translational potential of these findings based on key gene features. Overall, this study aims to dissect how microsatellite instability regulates the immune response in colorectal cancer at the clonal level, providing new insights for immune-biological stratification beyond traditional classification.

## 2. Result

### 2.1. Baseline TCR Repertoire Characteristics Predict Distinct Immune Response Landscapes

**Figure 1:**
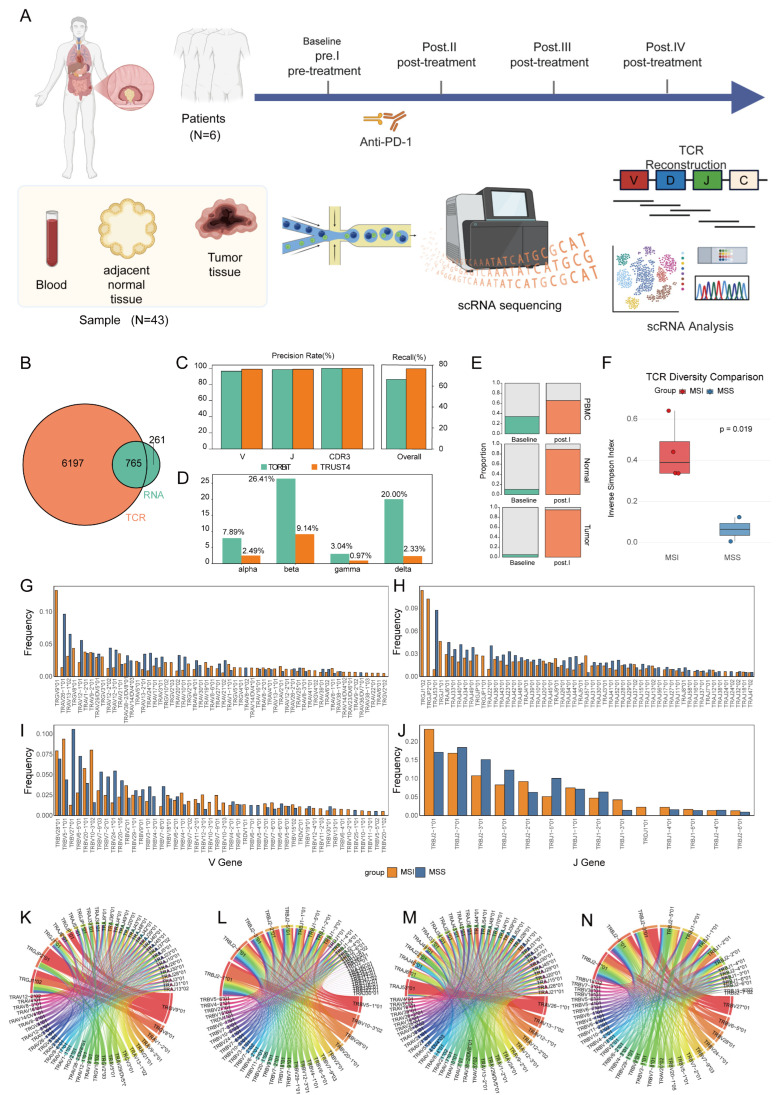
Data Overview and Baseline TCR Repertoire Characteristics Profiled by the TORBiT Pipeline. (**A**) Dynamic sample collection scheme and sequencing data types for six patients undergoing anti-PD-1 blockade therapy. (**B**) Number of complete TCR chains reconstructed from scRNA-seq and scTCR-seq data by our in-house pipeline, along with overlap counts. (**C**) Benchmark comparison of TORBiT against TRUST4. (**D**) The results assembled by TORBiT and TRUST4 are used to compare the full-length TCR sequences. (**E**) Clonotype expansion across different tissues in individual patients before and after treatment. (**F**) TCR clonotype expansion in MSI and MSS groups before and after treatment. (**G**–**J**) TCR gene segment usage under different microsatellite statuses. (**K**–**N**) Gene pairing patterns of the top 30 most frequent variable genes in TCR clonotypes.

To delineate the cellular composition and functional states of colorectal cancer under different conditions, we obtained single-cell RNA sequencing (scRNA-seq) and matched T-cell receptor sequencing (scTCR-seq) data for six patients who received neoadjuvant immunotherapy from the NGDC database. A total of 43 samples were collected across different tissues and time points, including blood (6 samples), adjacent normal tissue (5 samples), and tumor tissue (7 samples) obtained before and after treatment (Figure 1A). Among the MSS cohort, two patients (P01 and P02) achieved a complete response (CR) following treatment, whereas MSI patients showed partial response (PR) or no response (NR) to immunotherapy. Detailed sample information is provided in Supplementary Table S1.

To characterize the structural features of the TCR repertoire under different microsatellite statuses, we first developed and applied a TCR reconstruction analysis pipeline named “T cell Receptor Omics Reconstruction Bioinformatics Toolkit” (TROBiT), based on scRNA-seq data (Methods). This pipeline integrates transcriptomic information at the cellular level and reconstructs TCR sequences from scRNA-seq data through steps including sequence assembly, V(D)J annotation, and clonotype identification. To evaluate the reliability of the pipeline, we randomly selected one patient’s scRNA-seq sample as a test dataset and simultaneously assembled its matched scTCR-seq data to serve as a reference for authentic V(D)J sequences. The results showed that TROBiT assembled 6962 unique productive TCR contigs with complete V(D)J annotation from scTCR-seq data, and 1026 corresponding productive TCR contigs from scRNA-seq data, with 765 overlapping entries. The overlap rate was 10.99% relative to TCR-seq and 74.56% relative to RNA-seq (Figure 1A). Its assembly performance was comparable to that of the widely used tool TRUST4, which showed a TCR-seq overlap rate of 1.6% and an RNA-seq overlap rate of 70.7% [30]. Furthermore, we conducted a benchmark test of TORBiT’s gene recall rate and accuracy (Figure 1B). In terms of recall rate, TRUST4 demonstrated a higher overall recall (76.41% vs. 66.31%). Regarding precision, our tool achieved 96.48% for V gene annotation, 98.50% for J gene annotation, and 100% for CDR3 identification. In comparison, TRUST4 attained 98.99% for V gene annotation, 98.96% for J gene annotation, and 100% for CDR3 identification (Figure 1C). Notably, the full-length TCR sequence entries obtained by our tool are higher than those of TRUST4 (Figure 1D). These results validate the usability and stability of our pipeline for TCR reconstruction.

Subsequently, we applied this pipeline to 43 scRNA-seq samples from different tissues to systematically reconstruct TCR clonotypes. Specifically, a total of 189,096 blood-derived TCR clonotypes were identified in the MSI group (combined pre-and post-treatment samples), while 6428 and 9087 clonotypes were detected in normal and tumor tissues, respectively. In contrast, the MSS group exhibited 15,933, 4314, and 11,503 clonotypes in blood, normal tissue, and tumor tissue, respectively (Figure 1E and Figure S1). Additionally, we evaluated baseline peripheral blood TCR clonality in MSI and MSS patients prior to treatment. The results showed that the MSS group had a higher clonality score than the MSI group (*p* = 0.55), whereas the diversity score was significantly lower in the MSS group (*p* = 0.019) (Figure 1F and Figure S2B). These findings suggest that marked differences exist in the scale and compositional characteristics of the TCR repertoire between microsatellite-stable (MSS) and microsatellite-unstable (MSI) colorectal cancer patients.

The observed differences in TCR clonotype distribution and diversity across microsatellite statuses suggest that the TCR repertoire may follow distinct evolutionary trajectories in MSI versus MSS tumor microenvironments. By further quantifying TCR gene usage, we found clear preferential selection of genes between the MSI and MSS groups. In the MSI group, genes associated with γδ TCRs (e.g., TRGV9*01, TRGV8*01 (Figure 1G), TRGJ1*02, TRGJP2*01, and TRGJ*01) were utilized at higher frequencies and exhibited elevated expression levels (Figure 1H). Notably, the usage ratios of the δ-chain genes TRDV1*01 and TRDJ1*01, which pair with γ chains, were significantly lower (Figure 1I,J). This may be attributed to the multi-gene alignment strategy employed in our preliminary data analysis, which could have led to the misalignment of some δ-chain gene segments to α-chain regions (Figure 1G). In contrast, most genes in the MSS group displayed a co-expression pattern, with TRBV4-3*01 being unique to the MSS group, while TRBV27*01 and TRBV6-5*01 showed significantly higher expression in this group compared to the MSI group (Figure 1I). Regarding J gene usage, TRBJ2-7*01, TRBJ2-3*01, TRBJ2-5*01, and TRBJ1-5*01 exhibited only marginal expression advantages in the MSS group (Figure 1). Additionally, differences in gene pairing patterns were observed between the groups: the MSI group was dominated by clonotypes with TRGV9*01 paired with TRGJP2*01, and TRBV10-3*02 paired with TRBJ2-1*01, whereas the MSS group was primarily characterized by TRAV24*01 paired with TRAJ6*01, and TRBV27*01 paired with TRBJ2-3*01 (Figure 1K–N and Figure S2C–F). In summary, MSI and MSS tumors exhibit systematic differences in TCR clonal composition, V/J gene usage preferences, and chain pairing patterns. These findings suggest that microsatellite status drives the formation of fundamentally distinct TCR repertoire architectures by reshaping TCR gene selection and clonal expansion pathways, which may ultimately determine subsequent immune recognition and response patterns.

### 2.2. Characterization of Shared and Private TCR Clonotypes in MSI and MSS CRC

**Figure 2:**
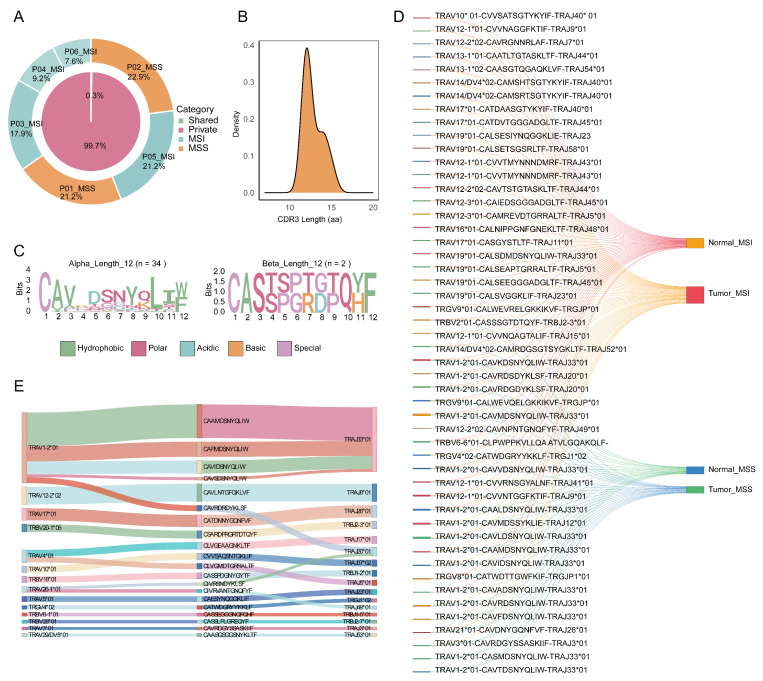
Cross-Patient Shared TCR Clonotypes Reveal Deterministic Selection Patterns. (**A**) Proportion of uniquely shared clonotypes among all clonotypes, and patient-specific proportions of unique clonotypes. The inner ring shows private versus shared proportions; the outer ring displays the percentage of each patient’s TCR clonotypes within the total TCR repertoire, grouped by MSI status. (**B**) CDR3 length distribution of the top 20 inter-group shared clonotypes. (**C**) Sequence logo of CDR3 amino acid sequences corresponding to the peak length in the CDR3 length distribution of the top 20 shared clonotypes. (**D**) Tissue sharing of the top 50 intra-group and inter-group shared clonotypes. Nodes on the left indicate the number of patients carrying a given clonotype (minimum 1, maximum 6); nodes on the right represent tissue types across different groups. A link between a tissue node and a clonotype indicates that the tissue shares that clonotype. (**E**) Gene selection and CDR3 amino acid sequence information for the top 20 inter-group shared clonotypes.

To further delineate the immune response landscape in colorectal cancer patients with different microsatellite statuses, we quantified the sharing architecture of the TCR repertoire by comparing the distribution of private, intra-group shared, and inter-group shared clonotypes. Private clonotypes predominated (99.7%), whereas inter-group shared clonotypes accounted for only 0.3% (Figure 2A). This distribution aligns with previous reports highlighting the high diversity of TCR repertoires [31]. Moreover, we observed that MSS patients achieving complete response (CR) (Patients 1 and 2) exhibited clonal richness comparable to that of MSI patients with partial response (PR), while MSI patients with no response (NR) (Patients 4 and 6) showed markedly lower clonal richness (Figure 2A). Analysis of inter-group shared clonotypes revealed a peak CDR3 amino acid length at 12 residues (Figure 2B and Figure S2A). Conservation analysis of CDR3 sequences with this predominant length identified highly conserved amino acid residues at specific positions (Figure 2C). These suggest that T-cell clones among different patients are not randomly generated but may have undergone selective pressures leading to convergent sequence patterns with shared structural constraints. On the other hand, comparing clonotype sharing between normal and tumor tissues (Figure 2D) revealed the presence of T-cell clones in adjacent normal tissue capable of recognizing tumor antigens and migrating to tumor sites. This indicates that both MSS and MSI colorectal cancer patients may harbor a potential immune surveillance network connecting normal and tumor tissues. Within this network, the MSI group displayed a greater diversity of shared clonotypes, consistent with the notion that MSI, as an “immunologically hot” tumor with higher mutational burden and neoantigen diversity, may activate a broader repertoire of T-cell clones. However, this quantitative advantage in clonotype diversity did not directly translate into superior clinical efficacy, prompting us to investigate the functional determinants of clones from a “quality” perspective. Further analysis of highly shared clonotypes revealed a pronounced bias in V-J gene segment pairing; for instance, the combination of TRAV1-2*01 with TRAJ33*01 was significantly more frequent than other pairings (Figure 2E). This finding reinforces the presence of a non-random, selective process, where the “commonality” of shared clones stems from preferential usage of specific TCR gene segments, upon which CDR3 diversity fine-tunes specificity. This pattern is characteristic of clonal convergence, often interpreted as indirect evidence for selection by common or structurally similar antigens. This mechanism also offers a potential explanation for the treatment responses observed in a subset of MSS patients.

### 2.3. Anti-PD-1 Therapy Induces Systemic Immune Cell Migration in Colorectal Cancer Patients

**Figure 3:**
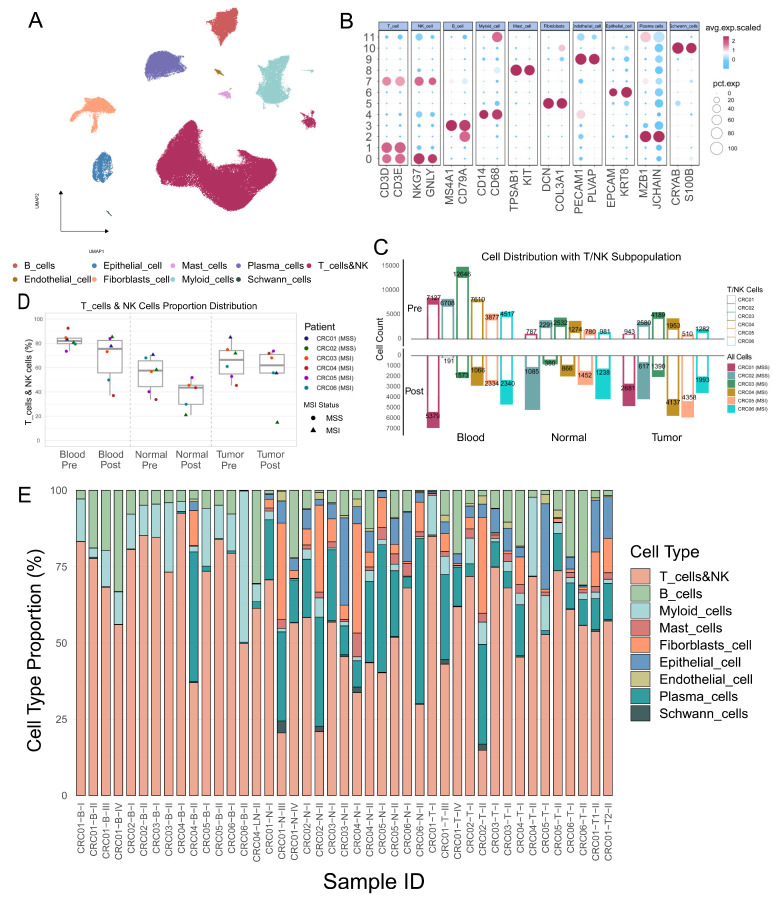
Single-Cell Atlas of Colorectal Cancer Patients with Different Microsatellite Statuses Undergoing Neoadjuvant Anti-PD-1 Therapy. (**A**) UMAP visualization of distinct cell-type populations. (**B**) Dot plot displaying expression markers for different cell types. (**C**) Total cell numbers per tissue across patients before and after treatment (Pre vs. Post), along with the distribution of T&NK cells across all cell clusters. (**D**) Expansion or contraction of T&NK cells in different tissues before and after treatment, stratified by microsatellite status. (**E**) Proportions of different cell types at each sampling time point for individual patients.

To extend the analysis of inter-tissue migration and T-cell functional state transitions observed in TCR clonal kinetics at the cellular population level, we collected single-cell transcriptome (scRNA-seq) data on the same set of samples. Following standardized quality control, a total of 175,930 high-quality cells were retained for downstream analysis. Nine major cell types were identified based on canonical marker genes: B cells (MS4A1, CD79A), endothelial cells (PECAM1, PLVAP), epithelial cells (EPCAM, KRT8), fibroblasts (DCN, COL3A1), mast cells (TPSAB1, KIT), Schwann cells (CRYAB, S100B), NK cells (NKG7, GNLY), plasma cells (MZB1, JCHAIN), and T&NK cells (CD3D, CD3E) (Figure 3A,B and Figure S3). We subsequently analyzed the cellular composition and proportional changes across different tissues in each patient. Multi-timepoint comparisons revealed a consistent pattern of immune cell remodeling across tissues following PD-1 blockade, regardless of microsatellite status. Specifically, although the absolute number of T&NK cells in the peripheral blood decreased in all patients (Figure 3C), the proportional changes in the blood exhibited inter-individual variability, with P02 and P05 showing an increase (Figure 3D,E). Concurrently, an upward trend in the proportion of T&NK cells was observed in the tumor tissue of some patients (P04, P05, P06), suggesting that this population may migrate from the periphery to the tumor site and participate in local immune responses. The discrepancy in T&NK cell proportions between peripheral blood and tumor tissue across patients further prompted us to investigate the underlying inter-individual heterogeneity in immune regulatory mechanisms.

### 2.4. Memory T-Cell Loss and Terminal Exhaustion Define the Immune Coercion Landscape in Microsatellite-Differing Colorectal Cancers

**Figure 4:**
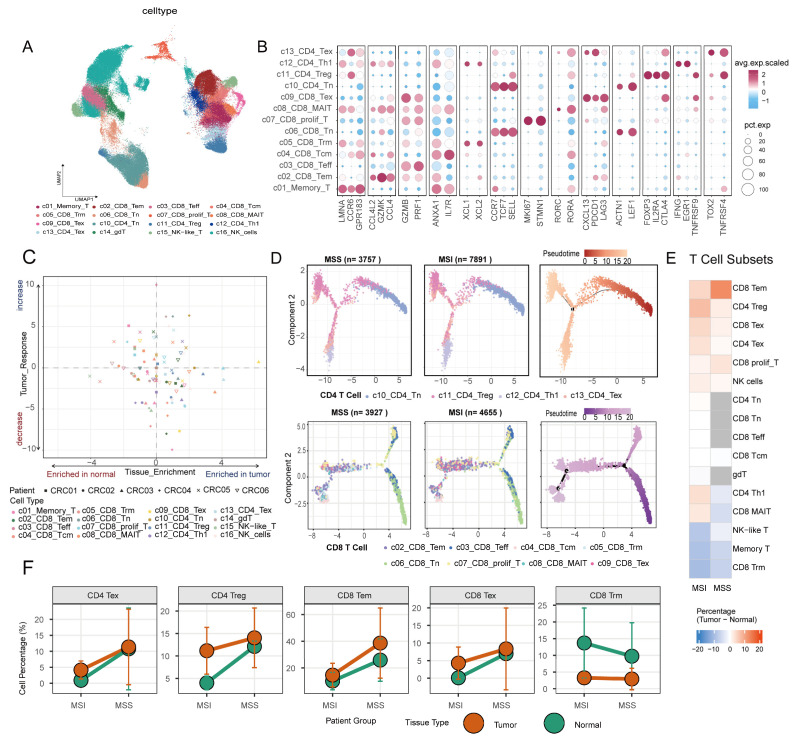
T-Cell Subset Classification, Tissue Enrichment, and Exhaustion Trajectories. (**A**) UMAP plot of distinct T-cell subsets. (**B**) Dot plot showing gene expression in CD4^+^ and CD8^+^ T-cell clusters. (**C**) Tissue-specific enrichment and dynamic changes in each T-cell subset in individual patients. (**D**) Pseudotime trajectory analysis for CD4^+^ and CD8^+^ T cells separately. (**E**) Heatmap of target T-cell subset proportion changes under different microsatellite statuses, calculated as the difference between post-treatment and baseline (pre-treatment) in tumor tissue minus the corresponding difference in normal tissue (ΔTumor−ΔNormal). (**F**) Dynamic changes in the proportions of target T-cell subsets stratified by microsatellite status, calculated as described above.

To further dissect the subset-specific dynamics and functional features of the observed inter-tissue migration of T&NK cells, we performed unsupervised clustering on T and NK cells, identifying 16 subsets. These included NK cells, NK-like T cells, γδ T cells, four CD4^+^ T-cell subsets, eight CD8^+^ T-cell subsets, and a subset of ambiguous memory-phenotype T cells (Figure 4A,B and Figure S4). We then evaluated the proportional changes in each T-cell subset before and after treatment (Figure 4C). Contrary to the prevailing view that MSS tumors are often considered “immune deserts” [32], we found that the adjacent normal tissue of MSS patients contained a higher proportion of effector-memory CD8^+^ T cells (c02_CD8_Tem) compared to MSI patients (Figure 4F), suggesting a potentially greater baseline immune reserve. Further analysis revealed that in MSS patients achieving complete response (CR), c02_CD8_Tem was significantly increased and enriched in tumor tissue after treatment (Figure 4C and Figure S5). More importantly, in CR patients (e.g., P02), the proportion of terminally exhausted CD8^+^ T cells (c09_CD8_Tex) did not increase post-treatment and was even significantly lower than baseline levels (Figure 4C and Figure S5), indicating that effective therapy is accompanied not only by expansion of effector cells but also by blockade of the terminal exhaustion process. In contrast, among MSI patients with no response (NR), we consistently observed elevated proportions of exhausted T cells (c09_CD8_Tex) and increased numbers of regulatory T cells (c11_CD4_Treg) (Figure 4E,F), suggesting that in some non-responders, exhaustion and immunosuppressive cell subsets may jointly contribute to the failure of immune responses. Pseudotime trajectory analysis of CD4^+^ and CD8^+^ T cells (Figure 4D) showed that although T cells generally exhibited a trend toward exhaustion, the terminal states of their trajectories differed between the two groups. Collectively, based on the dynamic analysis of T-cell subsets, we found that the core features of the colorectal cancer immune microenvironment are not solely determined by microsatellite instability status, but instead manifest as two distinct functional patterns: a “high-baseline, strong-suppression” phenotype in MSS tumors and a “high-fluctuation, deep-exhaustion” phenotype in MSI tumors. These distinct phenotypic landscapes set the stage for differential clonal dynamics, which we next investigated at the TCR repertoire level.

### 2.5. TCR Clonal Dynamics Confirm the “High-Fluctuation, Deep-Exhaustion” Immune Coercion Pattern in MSI Tumors

**Figure 5:**
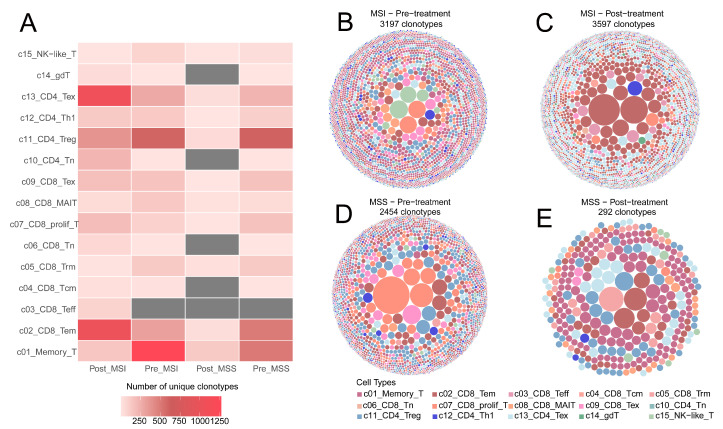
Distribution of T-Cell Subtype-Specific Clonotypes. (**A**) Changes in the number of unique clonotypes across different T-cell subtypes before and after treatment. (**B**) Panel of clonotype abundance for each T-cell subtype in the MSI group before treatment. (**C**) Panel of clonotype abundance for each T-cell subtype in the MSI group after treatment. (**D**) Panel of clonotype abundance for each T-cell subtype in the MSS group before treatment. (**E**) Panel of clonotype abundance for each T-cell subtype in the MSS group after treatment. In all panels (**B**–**E**), each circle represents a distinct clonotype; circle size corresponds to clonotype abundance, and color indicates different clonotypes.

Building on the distinct functional patterns between MSS and MSI patients revealed by the T-cell subset dynamics described above, we further compared TCR clonal dynamics before and after treatment to dissect the impact of microsatellite stability on the TCR repertoire at the clonal level. The results showed significant differences between the two groups in terms of clonal quantity changes and the remodeling patterns of functionally relevant subsets. In MSS patients, adjacent normal tissue harbored a sizable and diverse reservoir of TCR clones. However, within tumor tissue and during post-treatment dynamic changes, multiple functionally relevant T-cell clones in the MSS group displayed varying degrees of overall contraction. This included significant post-treatment declines in effector CD8^+^ T-cell clones (e.g., c02_CD8_Tem), exhaustion-associated CD8^+^ clones (c09_CD8_Tex), and regulatory T-cell clones (c11_CD4_Treg) (Figure 5A). This broad contraction, involving effector, exhausted, and immunoregulatory clones, led to an overall reduction in clonal abundance within the TCR repertoire of MSS patients after treatment (Figure 5D,E). In contrast, MSI patients exhibited more pronounced and directionally complex clonal changes. In the MSI group, multiple effector-associated clones (e.g., c02_CD8_Tem and c07_CD8_prolif_T) expanded markedly after treatment, indicating a higher capacity for T-cell activation and expansion. Concurrently, CD4^+^ exhaustion-associated clones (c13_CD4_Tex) in the MSI group also increased significantly, with an expansion magnitude exceeding that of most effector clones (Figure 5B,C). These findings demonstrate that the TCR repertoire of MSI patients exhibits a dual characteristic: synchronous expansion of effector clones and accumulation of exhausted clones before and after treatment.

### 2.6. T-Cell Functional State Balance Determines Prognosis in MSI-H Patients

**Figure 6:**
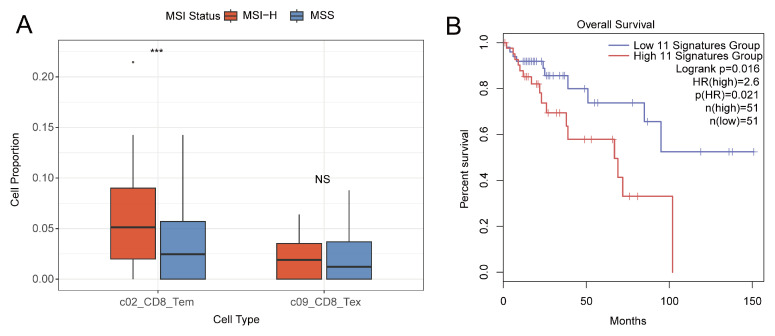
Validation of Cell Proportions and Prognostic Model in the TCGA Cohort. (**A**) Quantitative analysis of cell proportions in the TCGA dataset using CIBERSORT. Statistical significance: *** *p* < 0.001, and NS (not significant) denotes *p* ≥ 0.05. (**B**) Construction of a risk-prognostic model based on the 11 selected differentially expressed genes via GEPIA2.

To systematically evaluate the role of T-cell functional states in MSI colorectal cancer, we extended our analysis to a colorectal cancer cohort from The Cancer Genome Atlas (TCGA, n = 308), comprising 53 MSI-H and 255 MSS samples. We first performed immune cell infiltration quantification in the TCGA cohort. Using a reference matrix constructed from the key T-cell functional subsets defined in our single-cell data, we deconvoluted TCGA RNA-seq data. The results showed that the infiltration levels of multiple T-cell functional subsets—including CD8^+^ Tem, CD8^+^ Tex, and CD4^+^ Tex—were significantly higher in MSI-H tumors than in MSS tumors (Figure 6A and Figure S6). This observation aligns with the TCR dynamics we observed in single-cell data, confirming that MSI-H tumors exhibit an immune profile characterized by “high fluctuation and deep exhaustion”—encompassing both actively expanding effector clones and the accumulation of functionally impaired exhausted clones. Based on these findings, we further constructed a quantifiable survival risk model using 11 genes associated with T-cell functional maintenance (e.g., IL7R, GZMK) and exhaustion-driving pathways (e.g., NR4A1, HSPA1A). This model demonstrated good predictive performance for survival in an independent TCGA MSI cohort (n = 102). Kaplan–Meier analysis illustrated that the model could stratify patients into high- and low-risk groups based on individual risk scores (Figure 6B). High-risk scores corresponded to a gene-expression signature typical of a “deep-exhaustion” pattern, characterized by upregulation of exhaustion-related genes and downregulation of functional-maintenance genes. In contrast, low-risk patients exhibited an immune state closer to effector-function preservation. These results indicate that survival differences among MSI patients are primarily determined by the balance of T-cell functional states rather than merely by the overall quantity of T-cell infiltration.

## 3. Discussion

This study adopts a cross-tissue, longitudinal single-cell integrative framework that jointly analyzes scRNA-seq and scTCR-seq data to comparatively examine the tissue distribution, clonal evolution, and functional fate of T-cell responses in microsatellite-stable (MSS) and microsatellite-instable (MSI) colorectal cancers. In contrast to previous studies that primarily focused on a single tissue compartment or a single time point, this framework emphasizes the coordinated assessment of clonal connectivity and functional remodeling across peripheral blood, adjacent normal tissue, and tumor tissue in a treatment-relevant context. This holistic approach enables the differentiation of distinct immunodynamic mechanisms, including local expansion, peripheral recruitment, and functional transition. Within this analytical framework, we are able to elevate the understanding of T-cell immune state differences from static descriptions at the “compositional level” to a dynamic comprehension of “clonal selection and functional fate,” thereby providing a more explanatory analytical perspective for deciphering the differences in immunotherapy responses under different microsatellite statuses.

Our study shows that MSS colorectal cancer (CRC) exhibits a “high baseline-strong suppression” immune phenotype. Adjacent non-tumor tissues are enriched in memory CD8^+^ T cells (e.g., c05_CD8_Trm and c02_CD8_Tem), indicating substantial immune reserves, which is consistent with the concept that peritumoral tissues serve as immune cell reservoirs [33]. However, the tumor microenvironment is characterized by widespread accumulation of immunosuppressive cytokines and T-cell dysfunction [34,35]. Our TCR clonality analysis further revealed a global contraction of the TCR repertoire, high conservation of shared clones, and, notably, a failure of effector clones to expand—indeed, a decline—following PD-1 blockade. These clonal-level features provide a mechanistic explanation for the limited efficacy of PD-1 monotherapy in patients with MSS CRC.

In contrast, MSI CRC, driven by high mutational burden and abundant neoantigen generation, elicits robust T-cell recruitment and clonal expansion and is therefore more responsive to PD-1/PD-L1 inhibitors [13], accompanied by pronounced T-cell functional remodeling and exhaustion programs [36]. In our study, MSI tumors displayed significantly increased TCR diversity and dramatic expansion of effector CD8^+^ T-cell populations (e.g., c02_CD8_Tem and c07_CD8_prolif_T), reflecting sustained and intense immune activation. At the same time, terminally exhausted T cells (c13_CD4_Tex and c09_CD8_Tex) and Treg cells (c11_CD4_Treg) were markedly expanded, with the magnitude of exhausted clone expansion exceeding that of effector clones. This “high-fluctuation-deep exhaustion” clonal trajectory suggests that, although the MSI microenvironment possesses strong activating capacity, it can rapidly drive newly generated effector T cells into irreversible dysfunction. This provides a plausible explanation for primary resistance to PD-1 inhibitors in a subset of MSI patients: once T cells have entered terminal exhaustion under multiple suppressive cues, blockade of the PD-1 pathway alone may be insufficient to restore function [37,38]. Recent multi-omics studies have further identified DUB-H and DUB-L subtypes within MSS CRC based on the expression of immune-related deubiquitinating enzymes (IR-DUBs). The DUB-L subtype is characterized by higher immune infiltration, stronger T-cell inflammatory signatures, and improved relapse-free survival, whereas high USP7 expression is associated with immune desert and immunosuppressive states [18]. Collectively, these results underscore the intricate and heterogeneous nature of the colorectal cancer immune microenvironment, which cannot be adequately captured by a binary MSS/MSI-based classification into “cold” or “hot” tumors. A more refined molecular and functional stratification is therefore necessary, and our analytical approach offers a robust and scalable framework to achieve this goal.

Based on these findings, we constructed a prognostically relevant T-cell functional-state signature and validated it in the TCGA cohort. This signature integrates multiple key genes involved in T-cell exhaustion and functional regulation, including markers reflecting cellular stress and dysfunction. Notably, NR4A1 was prominently upregulated in high-risk patients; this transcription factor has been well documented to drive T-cell exhaustion and impair effector function [39]. CXCR4 has been shown to promote metastasis and immunosuppression in colorectal cancer [40], while CCL4 can recruit regulatory T cells and myeloid-derived suppressor cells, contributing to the formation of an immunosuppressive tumor microenvironment [41]. The expression pattern of this signature is characterized by elevated exhaustion/stress markers together with suppressed memory and effector potential (e.g., reduced expression of IL7R and GZMK), and it outperformed MSI status alone in stratifying patient survival. Our model suggests that combinatorial interventions targeting exhaustion pathways (such as NR4A1), or strategies aimed at enhancing T-cell persistence to improve functional fitness, may help overcome immunotherapy resistance in MSI colorectal cancer.

Despite the high-resolution insights gained from our spatiotemporal integrative framework, this study has several limitations that warrant awareness. First, the primary discovery cohort consists of a relatively small number of patients (n = 6). Although we leveraged a dense sampling strategy—analyzing 43 longitudinal specimens across multiple tissue compartments—the limited “n” per microsatellite subtype (MSI vs. MSS) may restrict the generalizability of certain rare T-cell clonal trajectories. We consider this work a pilot study that establishes a novel “clonal fate-centered” paradigm rather than a definitive clinical census. Second, while our findings on T-cell exhaustion and clonal contraction were robustly validated in the large-scale TCGA cohort, the lack of an independent, longitudinal single-cell validation set remains a constraint. Future studies involving larger multi-center cohorts will be essential to confirm these immunodynamic patterns across diverse treatment regimens. Nevertheless, our study provides a scalable analytical framework and offers a pioneering perspective on how clonal evolution, rather than static composition, dictates immunotherapy outcomes in colorectal cancer.

In summary, this study developed and applied TORBiT, an in-house toolkit for high-accuracy reconstruction of full-length TCRs from scRNA data, and through integrative analysis of cross-tissue, longitudinal single-cell and TCR clonotype data, revealed fundamental differences in T-cell immune evolutionary trajectories between MSI and MSS colorectal cancers. MSS tumors display a “strongly suppressive” phenotype characterized by clonal contraction and functional silencing, whereas MSI tumors follow a “high-fluctuation, deep-exhaustion” trajectory in which intense immune activation develops in parallel with terminal exhaustion programs. Molecular features derived from T-cell functional states indicate that patient prognosis is primarily determined by T-cell functional quality rather than the magnitude of immune infiltration, suggesting that reliance on MSI status or conventional “hot/cold” classifications alone may be insufficient to capture the biological basis of immune responses. Overall, this study proposes a T-cell clonal fate-centered analytical paradigm, providing a scalable framework for dissecting immune heterogeneity in colorectal cancer at both single-cell and clonal levels.

## 4. Methods

### 4.1. Data Collection

To characterize the cellular composition and functional states in colorectal cancer under microsatellite-stable (MSS) and microsatellite-instable (MSI) conditions, data were obtained from the National Genomics Data Center (NGDC) under accession number GSA HRA005546. The author team obtained permission for in-depth analysis of these data through a collaborative agreement. Library construction (10× Genomics 5′ V(D)J and 3′ RNA-seq) and sequencing (Illumina NovaSeq 6000, 150 bp paired-end reads) were performed by the original research group following the manufacturer’s standard protocols. Specifically, all samples were processed using the 10× Chromium platform (10× Genomics, Pleasanton, CA, USA) for both 3′ RNA-seq and V(D)J enrichment library preparation. The purified libraries were subsequently sequenced on the Illumina NovaSeq platform (Illumina, San Diego, CA, USA) with 150 bp paired-end reads. Read alignment and initial expression matrix generation were performed by the original research group using the Cell Ranger single-cell toolkit (10× Genomics, Pleasanton, CA, USA; version 6.1.2) with the GRCh38 human reference genome. The raw single-cell RNA sequencing data described above are publicly available through the GEO database under accession number GSE236581. After standardized quality control, we retained a total of 175,930 high-quality cells for downstream analysis. Detailed sample information is provided in Supplementary Table S1.

To extend our findings from the single-cell level to a larger cohort and assess their clinical relevance, we further integrated bulk transcriptomic data from The Cancer Genome Atlas (TCGA) database (https://www.cancer.gov/ccg/research/genome-sequencing/tcga; accessed on 25 October 2025). Specifically, transcriptomic and clinical metadata from the TCGA-COAD and TCGA-READ projects were retrieved. A rigorous preprocessing pipeline was implemented to ensure data integrity: transcriptomic profiles were cross-matched with clinical metadata using unique sample identifiers, retaining only patients with both high-quality expression profiles and confirmed microsatellite instability (MSI) status. Based on the clinical MSI_group information, samples were stratified into MSI-high (MSI-H, n = 53) and microsatellite-stable (MSS, n = 255) groups, while patients with ambiguous or missing clinical labels were excluded. This resulted in a finalized validation cohort of 308 samples. For downstream analysis, raw expression counts were converted to Transcripts Per Million (TPM) and log-transformed (log_2_(TPM + 1)) to eliminate sequencing depth bias and optimize the data distribution for immune deconvolution and survival modeling.

### 4.2. scRNA-Seq Data Processing

scRNA-seq data were analyzed using the R package Seurat (version 4.4.1). Rigorous quality control was performed to filter out low-quality cells based on two criteria: the number of detected genes and the proportion of mitochondrial gene counts. Specifically, cells meeting either of the following conditions were removed: (1) fewer than 200 or more than 6000 detected genes, or (2) mitochondrial gene content exceeding 5%. The NormalizeData function was applied for library-size correction and log-transformation, and the resulting expression matrix was used for downstream analysis.

### 4.3. Integration, Unsupervised Dimensionality Reduction, Clustering, and Cell Type Identification of Single-Cell Sequencing Data

Subsequently, we adapted the Seurat workflow to perform dimensionality reduction and unsupervised clustering. First, 2000 highly variable genes (HVGs) were selected using the FindVariableFeatures function with the parameter selection.method = “vst”. Next, the effects of total UMI counts and mitochondrial gene percentage were regressed out from the HVG expression matrix using the ScaleData function. Dimensionality reduction was then performed on the scRNA-seq data via the RunPCA function. Since our samples were collected from blood, adjacent normal tissue, and tumor tissue at multiple time points before and after anti-PD-1 immunotherapy and were processed in batches, we applied RunHarmony from the Harmony package (version 1.2.4) to identify anchors, perform integration, and remove batch effects. Principal components (PCs) were selected by ranking them using the ElbowPlot function in Seurat, which randomly permutes subsets of the data and computes projected PCA scores. When the elbow point was reached at the 30th principal component, the first 30 PCs were used for UMAP (Uniform Manifold Approximation and Projection) analysis via the RunUMAP function. Subsequently, the single-cell landscape was visualized by applying the FindClusters function with a resolution of 0.1. Cell clusters were then annotated based on canonical marker genes. For T-cell subpopulation clustering, the resolution was increased to 2, while all other parameters remained unchanged.

### 4.4. T-Cell Subtype Enrichment and Expansion

To quantitatively assess T-cell dynamic changes induced by anti-PD-1 therapy under different microsatellite statuses in colorectal cancer, we analyzed the distribution differences in T-cell subtypes between the tumor microenvironment and peripheral normal tissues, as well as changes in clonal size before and after treatment. Calculations were based on single-cell data from each patient at baseline (pre-treatment) and the first post-treatment sampling.

Let Tpre denote the abundance of a given T-cell subtype in tumor tissue at the first sampling time point (pre-treatment), and Npre denote the corresponding abundance in normal tissue at the same pre-treatment time point.

Tissue Enrichment Score:

This score quantifies the inherent distribution preference of a specific T-cell subtype between tumor and normal tissues. It is calculated as follows:

$$E=\log_{2}\left(\frac{T_{\mathrm{pre}}}{N_{\mathrm{pre}}}\right).$$

E > 0: indicates enrichment of the T-cell subtype in tumor tissue.

E < 0: indicates enrichment of the T-cell subtype in normal tissue.

Tumor Response Score:

This index measures the change in clonal size of a specific T-cell subtype after treatment relative to baseline. It is calculated as follows:

$$\Delta =\log_{2}\left(\frac{T_{\mathrm{post}}}{T_{\mathrm{pre}}}\right)$$

Δ > 0: indicates relative expansion of the T-cell subtype after treatment.

Δ < 0: indicates relative contraction of the T-cell subtype after treatment.

### 4.5. TCR Reconstruction Pipeline Design

For raw sequencing reads, alignment was first performed using BWA (version 0.7.18) [42] to filter reads originating from TCR regions. The alignment results were then converted to FASTQ format using samtools (version 1.17) [43]. For bulk sequencing data, the assembly module Trinity (version 2.1.1) [44] was directly invoked for batch processing. For single-cell sequencing data, a barcode-embedding strategy was introduced to enable single-cell-level parsing and demultiplexing, as follows: (1) Single-end strategy: Based on the characteristic that single-end reads share the same sequencing identifier, barcode information was embedded into the sequence ID, and clustering was performed according to the newly generated names to achieve single-cell-level splitting. (2) Paired-end strategy: For paired-end reads, barcode information was embedded into the IDs of both forward (F) and reverse (R) reads, followed by barcode-based clustering and splitting. To improve processing efficiency, multi-process parallelization was employed, and the assembled contigs were consolidated into a new FASTA file. Finally, functional annotation was carried out using the standalone annotation module of TRUST4 (version 1.1.5) [29] to extract complete TCR sequences. The code TORBiT is available at https://github.com/XieBioLab/TORBiT (accessed on 1 December 2025).

### 4.6. TCR Reconstruction Pipeline Evaluation

TCR information for each cell was reconstructed separately from single-cell TCR sequencing (scTCR-seq) and single-cell RNA sequencing (scRNA-seq) data. We defined the number of TCR chains reconstructed from scTCR-seq data as the benchmark. When a TCR chain reconstructed from scRNA-seq data for a given cell was successfully matched to a corresponding chain in the scTCR-seq benchmark, it was considered a correct TCR ligand for that cell; otherwise, it was classified as incorrect. The accuracy rate was calculated as the proportion of correctly reconstructed TCR chains relative to the total number of TCR chains, using the following formula:

$$X=\left(\frac{{ΤP}_{\mathrm{RNA}}}{{ΤP}_{\mathrm{RNA}}+{ΤN}_{\mathrm{RNA}}}\right).$$

To evaluate the performance of our TCR identification pipeline, we conducted a comprehensive benchmark comparison against TRUST4, a widely used tool for TCR sequence analysis. The evaluation was performed on a single-cell RNA sequencing dataset containing 6,614,682 raw sequencing reads. Both tools processed the same input data, and their outputs were systematically compared using two key metrics: recall rate and precision rate. Recall rate (sensitivity) was calculated as the proportion of original reads successfully identified and clustered by each tool, defined as: Recall rate = (Number of identified reads/Total original reads) × 100%. Precision rate (positive predictive value) was calculated as the proportion of identified reads successfully annotated as genuine TCR sequences, evaluated for each TCR component (V, D, J genes and CDR3 region): Precision rate = (Number of true positive reads/Total identified reads) × 100%. For each tool, we quantified: (1) the number of clustered sequences after the initial alignment step, (2) the number of sequences that obtained successful TCR annotations, and (3) the annotation success rates for V, D, J genes and the CDR3 region. Special emphasis was placed on evaluating D-gene annotation capability.

### 4.7. TCR Chain Filtering Criteria

Contigs assembled by our pipeline were filtered to select T-cell receptor (TCR) sequences, with the integrity and conservation of the CDR3 region serving as the core filtering criteria. To ensure analytical accuracy and biological relevance, only TCR chains containing complete variable region gene segments were retained as valid sequences for downstream analyses. Specifically, for T-cell receptor α (TRA) and γ (TRG) chains, a CDR3 amino acid sequence was considered valid if it started with a cysteine (C) and ended with either tryptophan (W) or phenylalanine (F). This criterion captures the typical C…F/W terminal pattern of CDR3 regions in these subtypes, consistent with the conserved features encoded by their J-region genes. For the more stringently conserved T-cell receptor β (TRB) and δ (TRD) chains, the CDR3 sequence was required to begin with the highly conserved “CASS” motif (cysteine-alanine-serine-serine) and end with phenylalanine (F) [45,46,47,48].

### 4.8. Individual Clonotype Expansion Proportion

Data from the same tissue of the same patient at different time points were integrated. The total number of clonotypes across both time points was set as 100%, and the proportion of clonotypes at each time point was calculated separately to assess induced expansion or contraction following treatment. Overall changes in clonal composition were evaluated by comparing the distribution of clonal frequencies (Ri) across all clonotypes, and further quantified using statistical measures such as the Clonal Expansion Index (CEI):

$$\mathrm{CEI}=\frac{1}{N}\sum_{i=1}^{N}R_{i}.$$

Peripheral Blood Baseline TCR Repertoire Clonality and Diversity Assessment:

To quantitatively assess differences in the clonal structure of the peripheral blood TCR repertoire between MSI and MSS colorectal cancer patients before receiving anti-PD-1 therapy, we performed the following analyses on pre-treatment (baseline) peripheral blood samples: Full-length TCR sequences were reconstructed from scRNA-seq data of each patient’s pre-treatment peripheral blood sample, and information including clonal frequency (Clones), CDR3 amino acid sequence (CDR3.aa), and V/D/J gene usage was recorded for each TCR sequence. Data cleaning and format standardization were carried out using a custom R script (extended from the immunarch package) to ensure the completeness and reliability of the clonotype list for each sample.

Clonality Score Calculation:

The clonality score was defined as the cumulative frequency of the top 10% high-frequency clones (ranked by Clones) relative to the total number of clones in the sample. The formula is as follows:

$$\mathrm{Clonality}=\frac{\sum_{i=1}^{k}{Clones}_{i}}{Total\_Clones},$$

where k = max(1,[0.1 × n]), and n denotes the number of unique clonotypes in the sample. This metric reflects the concentration of dominant clones within the TCR repertoire.

Diversity Score Calculation:

The diversity score was estimated as the ratio of clonotype richness (Unique Clones) to the total number of clones (Total Clones), i.e.,

$$\mathrm{Diversity}=\frac{Unique\_Clones}{Total\_Clones}.$$

A higher value of this ratio indicates a more even distribution of clonotypes within the TCR repertoire, reflecting greater diversity. Differences in clonality and diversity scores between the MSS and MSI groups were compared using independent two-sample *t*-tests, with a significance level set at *p* < 0.05. All calculations and visualizations were performed in the R environment (version 4.5.1), using ggplot2 for plotting and ggpubr for adding statistical annotations.

### 4.9. Private and Shared Clonotypes

Clonotype sharing was defined as identity in both variable gene selection and CDR3 amino acid sequence for either the α- or β-chain. A clonotype present in only one patient was classified as private. If a clonotype was found in multiple patients within either the MSI or MSS group, it was termed intra-group shared; if it appeared across both MSI and MSS groups, it was designated inter-group shared. To visualize the tissue distribution of highly shared T-cell clonotypes, we constructed Sankey diagrams based on single-cell TCR sequencing data. First, clonotypes present in both normal tissue (Normal) and tumor tissue (Tumor) were selected from the complete clonotype dataset, and the total number of patients carrying each clonotype, as well as the patient count stratified by MSI status, were summarized. The top 50 clonotypes observed in the largest number of patients were selected for further analysis.

### 4.10. Statistical Methods

Statistical analyses in this study were primarily performed using R (version 4.3.1) and associated packages. A significance threshold of *p* < 0.05 was applied for all statistical tests, and multiple-comparison corrections were conducted using the false discovery rate (FDR) method.

Comparison of continuous variables: For comparisons of continuous variables between two groups—such as immune cell proportions, TCR clonality scores, and diversity scores—independent two-sample *t*-tests were used when data met assumptions of normality and homogeneity of variances; otherwise, the Mann–Whitney U test (Wilcoxon rank-sum test) was applied. For comparisons among more than two groups, the Kruskal–Wallis test was employed. All box plots presented in the study (e.g., Figure 6A and Figure S5) were compared using the Wilcoxon rank-sum test.

Survival analysis: In the validation cohort, survival curves were plotted using the Kaplan–Meier method, and differences between groups were assessed with the log-rank test. The Cox proportional hazards model was used to calculate hazard ratios (HRs) and their 95% confidence intervals to evaluate the independent predictive value of the prognostic risk score.

Correlation analysis: Associations between gene expression and immune cell infiltration levels were evaluated using Spearman’s rank correlation analysis.

Significance levels in figures are denoted as follows: * *p* < 0.05, ** *p* < 0.01, *** *p* < 0.001. NS indicates no statistical significance.

### 4.11. Prognostic Risk Model Construction

To validate the prognostic value of T-cell functional states in an independent cohort, we constructed a multi-gene risk-scoring model based on 11 T-cell function-related signature genes identified from single-cell analysis (NR4A1, GZMK, HSPA1A, OASL, CXCR4, CCL4, ENC1, DUSP2, IL7R, PRKCQ-AS1, MATK). Using the GEPIA2 online platform, the model was built separately for MSI-H and MSI-L patient subgroups within the TCGA colorectal cancer cohort. Patients were dichotomized into high-risk and low-risk groups based on the median risk score. Prognostic performance was evaluated using Kaplan–Meier survival analysis and Cox regression embedded in the platform. The model demonstrated significant survival discrimination in the MSI-H subgroup (Log-rank *p* = 0.016).

## Data Availability

The single-cell transcriptome analyzed in this study was obtained from the Gene Expression Omnibus (GEO) database under accession number GSE236581. The code TORBiT is available at: https://github.com/XieBioLab/TORBiT, accessed on 25 October 2025.

## Supplementary Materials

The following supporting information can be downloaded at: https://www.mdpi.com/article/10.3390/ijms27062689/s1.
