# Supplementary material for: Single-Cell Multi-Tissue T Cell Clonal Dynamics Reveal Distinct Immune Coercion Landscapes in MSI and MSS Colorectal Cancer
Source: Int J Mol Sci. 2026 Mar 16;27(6):2689. doi: 10.3390/ijms27062689 (PMC13027115; doi:10.3390/ijms27062689)
Supplement: Supplementary file 1 [file ijms-27-02689-s001.zip › ijms-4169871-supplementary.pdf]

SUPPLEMENTARY MATERIAL

Supplementary Table S1. Sample Information: Age, Gender, Tumor stage, Tumor Location, Response, Tissue, Sampling Time Point, and Microsatellite Status

Patient	MSI statu	Age	Gender	Tumor stage	Tumor Location	Response	Tissue	Timepoint
P01	MSS	51	Male	II	Descend ing	CR	Blood<br>Norma<br>Tumor	I, II, III, IV<br>I,III,IV<br>I,II-1,II-2,III,IV
P02	MSS	64	Male	III	Low rectum	CR	Blood<br>Norma<br>Tumor	I, II<br>I , II<br>I, II
P03	MSI	60	Male	III	Sigmoid	PR	Blood<br>Norma<br>Tumor	I, II<br>I, II<br>I, II
P04	MSI	64	Male	III	Low rectum	NR	Blood<br>Norma<br>Tumor<br>LN	I, II<br>I, II<br>I, II<br>II
P05	MSI	37	Male	III	Sigmoid colon	PR	Blood<br>Norma<br>Tumor	I, II<br>I, II<br>I, II
P06	MSI	70	Male	IV	Ascendi ng colon	NR	Blood<br>Norma<br>Tumor	I, II<br>I, II<br>I, II

Supplement Figures

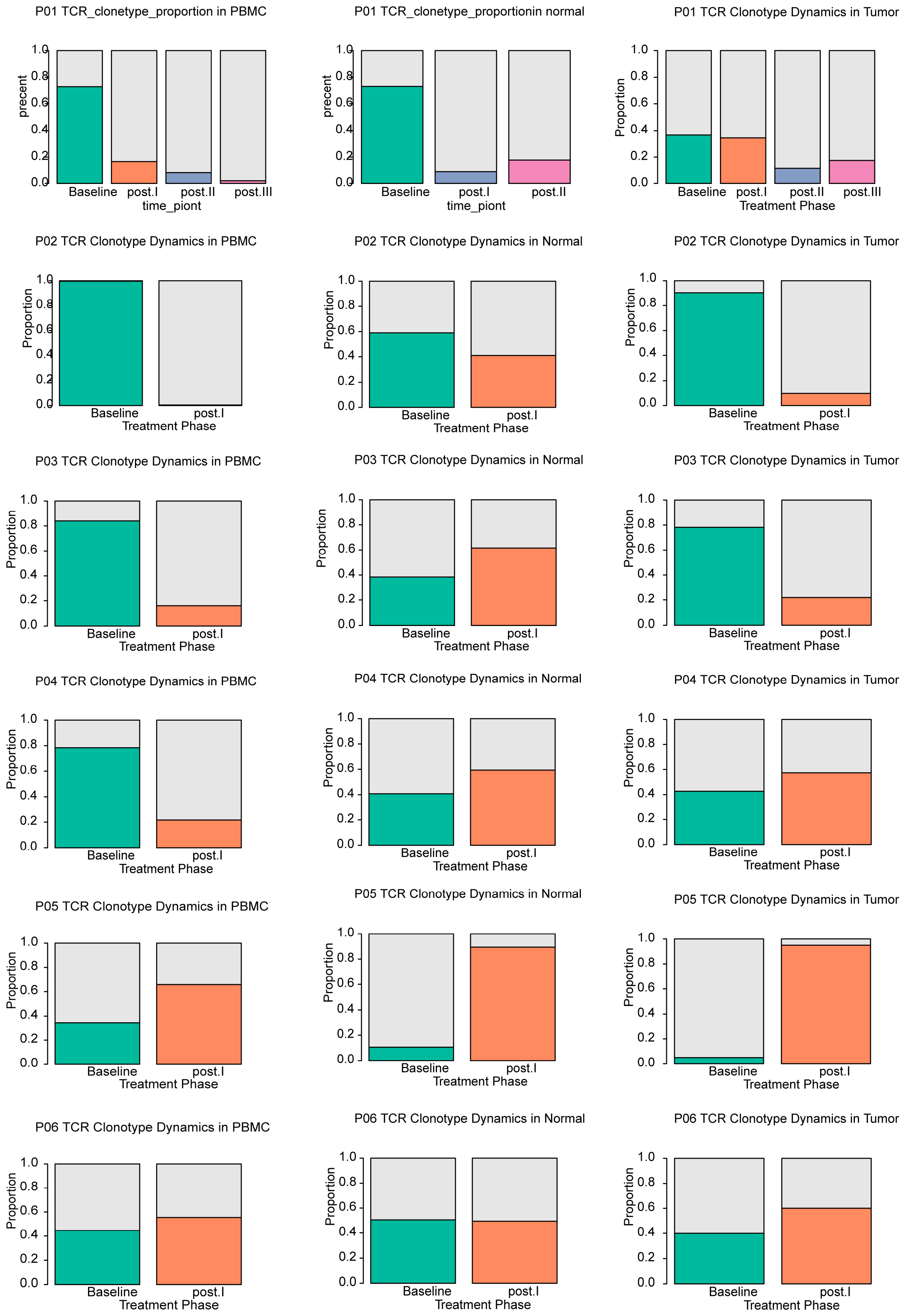

Supplemental Figure S1. Dynamic Changes of TCR Repertoires in T Cells Across Peripheral Blood, Normal Tissue, and Tumor Tissue for Each Patient

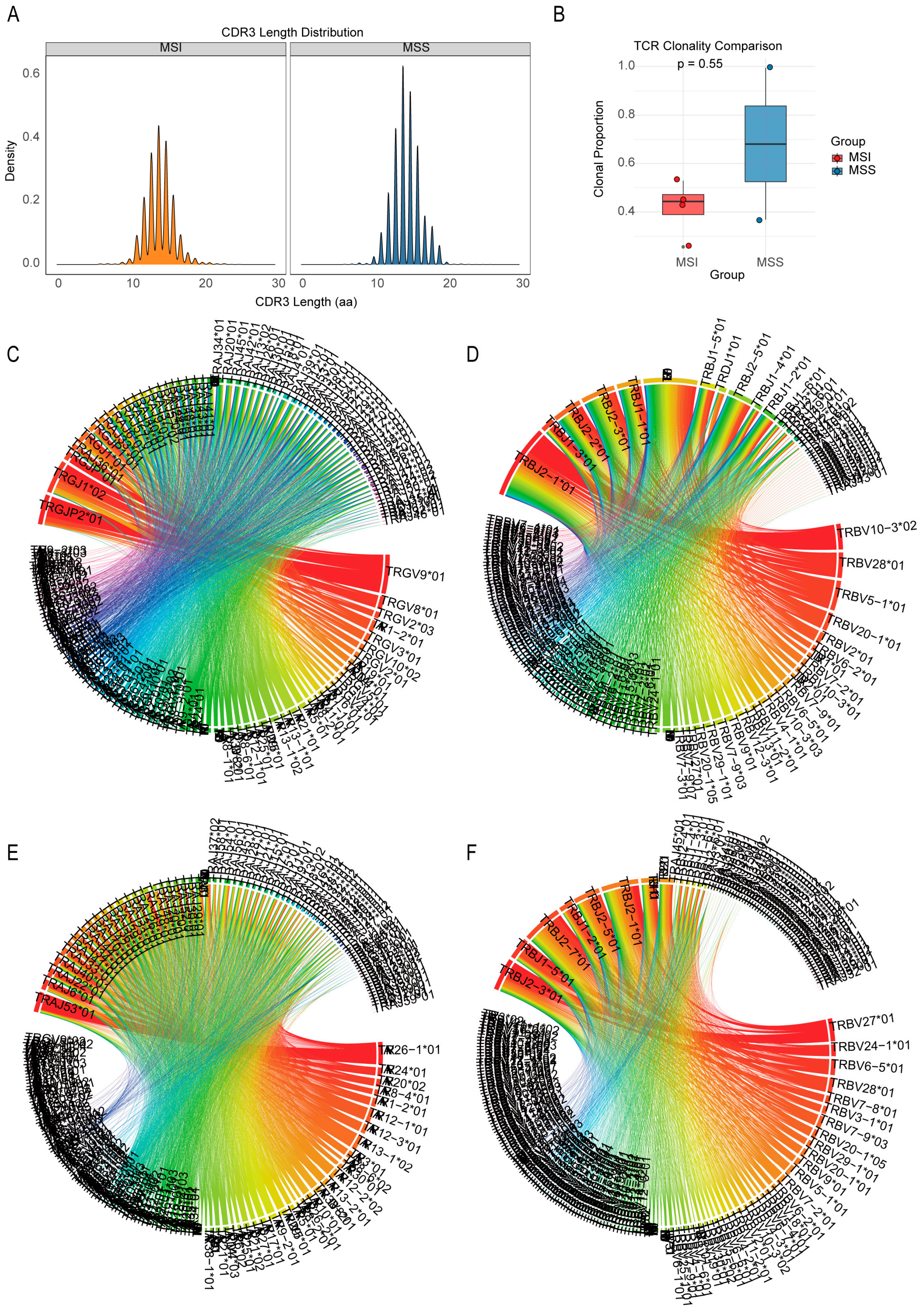

Supplemental Figure S2. **A.**CDR3 Length Distribution in MSI and MSS Groups; **B.** TCR clonality Comparison among different groups. **(C-F)** Gene pairing patterns in the variable regions of TCR clonotypes.

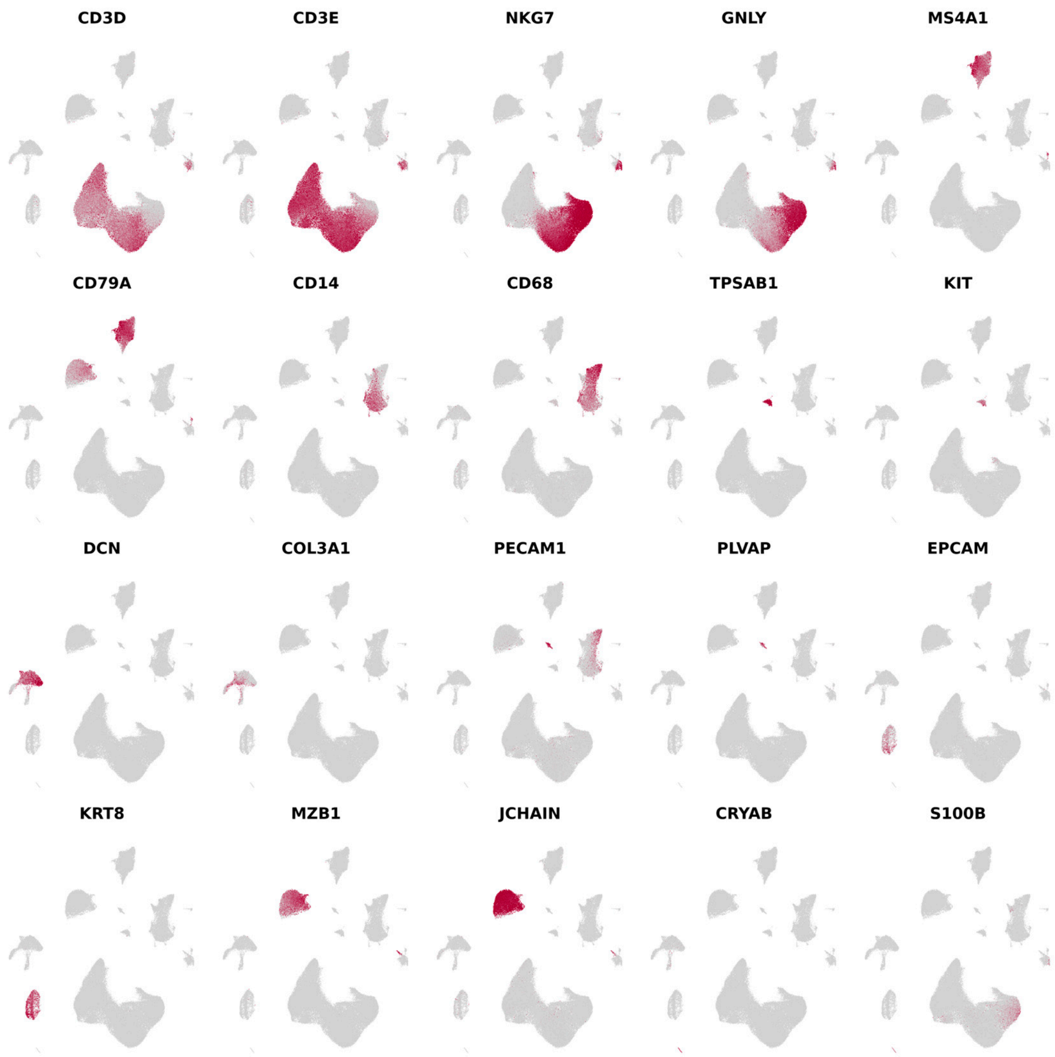

Supplemental Figure S3. Gene Signature of Major Cell Types

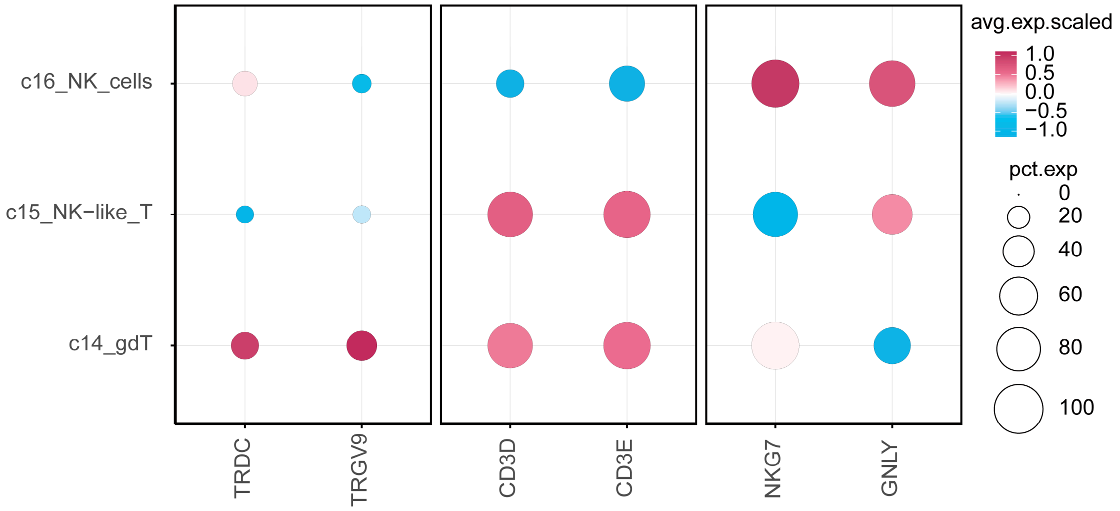

Supplemental Figure S4. Marker Gene Expression Plot for Distinguishing NK Cells and NK-like T Cells

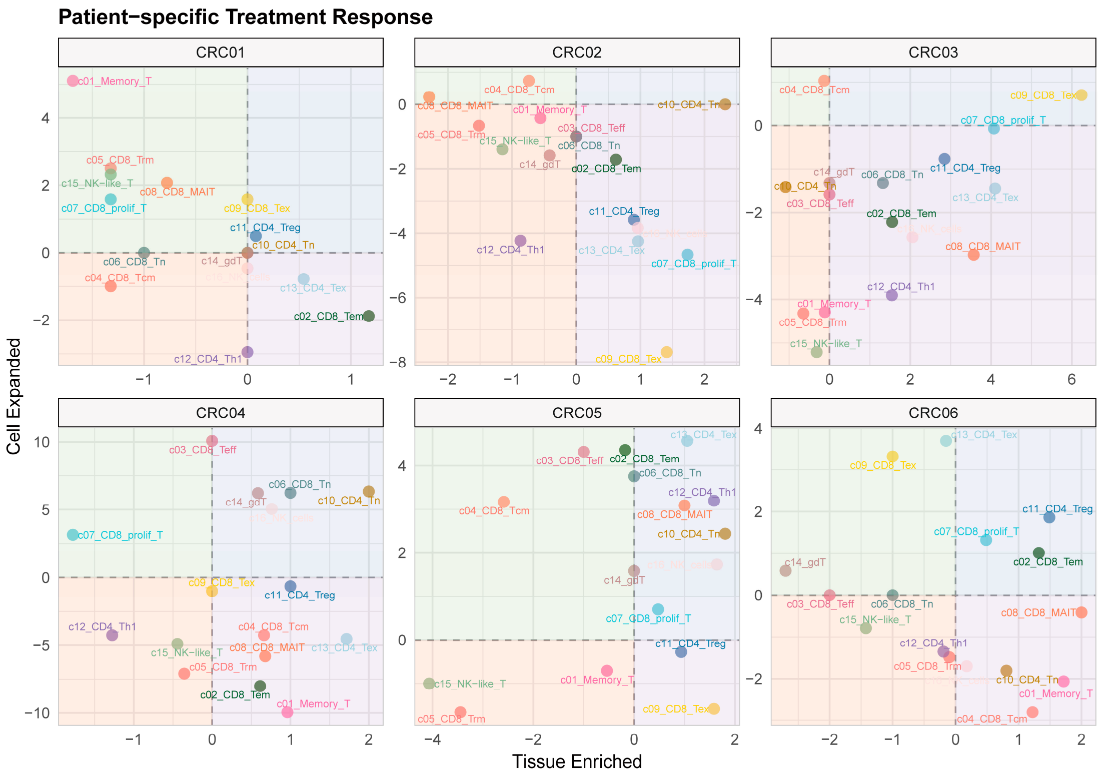

Supplemental Figure S5. Cell-Type Enrichment Status and Expansion Patterns Before and After Treatment for Each Patient

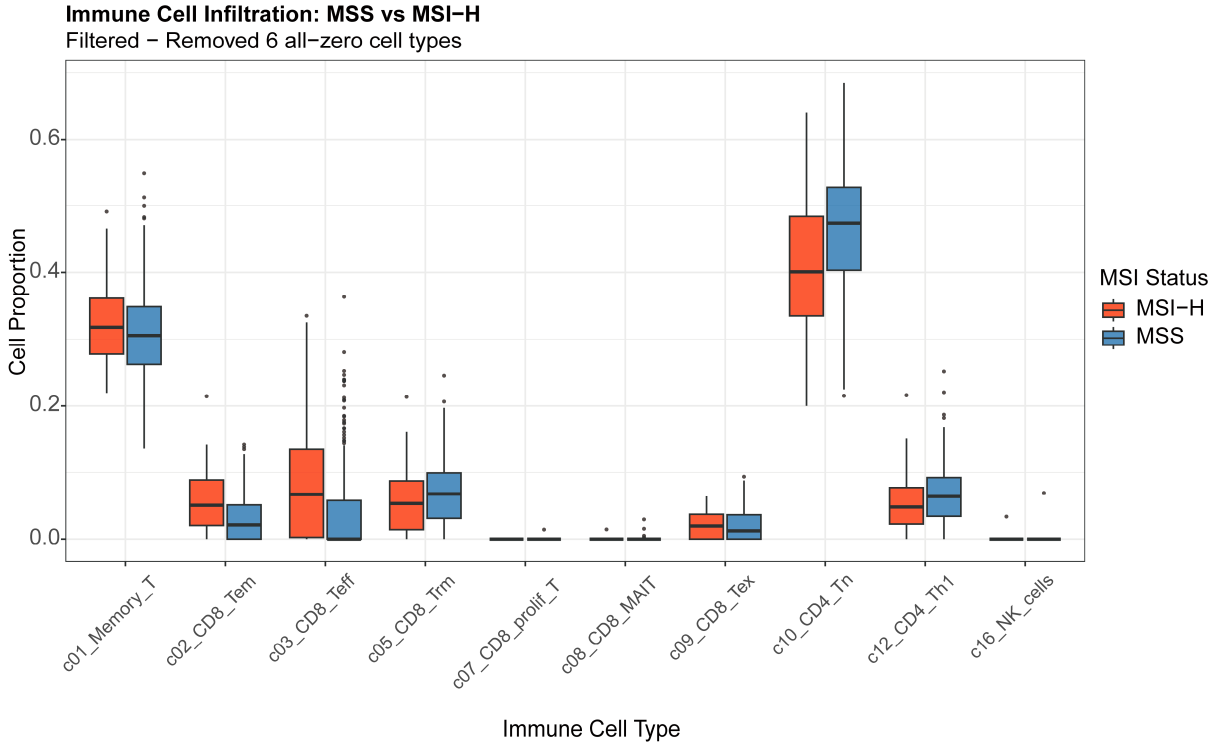

Supplemental Figure S6. Visualization of All Cell Subtypes in TCGA Validation of the 'High-Fluctuation, Deep-Exhaustion' Profile in MSI Tumors
